# Ten tips on how to prevent and manage post-transplant lymphoproliferative disease in kidney transplant recipients

**DOI:** 10.1093/ckj/sfag088

**Published:** 2026-03-14

**Authors:** Rose Mary Attieh, Armin Ghobadi, Naoka Murakami

**Affiliations:** Department of Transplant, Mayo Clinic Jacksonville, FL, USA; Division of Oncology, Washington University in St. Louis, Saint Louis, MO, USA; Division of Nephrology, Washington University in St. Louis, Saint Louis, MO, USA

**Keywords:** cancer, graft survival, immunosuppression, kidney transplantation, mycophenolate mofetil, nephrotoxicity

## Abstract

Post-transplant lymphoproliferative disease (PTLD) remains a challenging and high-impact complication of kidney transplantation, requiring thoughtful prevention, timely recognition, and multidisciplinary management. Key strategies include careful selection of immunosuppressive regimens, particularly avoiding belatacept in Epstein–Barr virus (EBV)-seronegative recipients and exercising caution with lymphodepleting induction in EBV-mismatched pairs, along with targeted EBV surveillance in high-risk patients. Once PTLD is suspected or confirmed, accurate staging and a stepwise treatment approach beginning with reduction of immunosuppression and escalating to rituximab, chemotherapy, or advanced cellular therapies are essential, with EBV-CTLs and chimeric antigen receptor T-cell therapy offering promising options in refractory disease. Close monitoring for infectious complications, cytopenias, and rejection is critical throughout treatment. Importantly, a prior history of PTLD should not preclude future transplantation in appropriately selected patients with sustained remission. Continued research is needed to refine surveillance strategies, clarify optimal treatment sequencing, and improve long-term outcomes for kidney transplant recipients at risk for or affected by PTLD.

## INTRODUCTION

**Figure 1:**
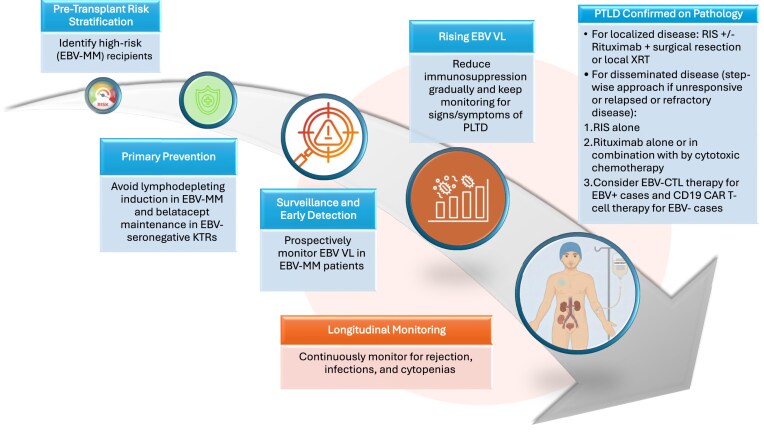
Stepwise approach for the prevention and management of PTLD in KTRs. EBV-MM, EBV mismatch; XRT, radiation therapy.

PTLD is a devastating complication of kidney transplantation, encompassing a wide histopathological spectrum with distinct clinical phenotypes. Its incidence follows a bimodal distribution: early-onset PTLD, occurring within the first 1–2 years post-transplant, is attributed to EBV infection in >90% of cases, whereas late-onset PTLD, typically developing 7–10 years after transplant, is more often EBV-negative [1, 2]. EBV mismatch (donor seropositive/recipient seronegative or D+/R−) is the strongest known risk factor, conferring a 5- to 10-fold higher incidence of PTLD compared with EBV-seropositive recipients, with rates of 7%–22% reported in recent studies [3, 4]. Management requires tight coordination between nephrologists and oncologists, with treatment plans tailored to the patient’s age, comorbidities, disease stage and histologic subtype, allograft function, immunosuppressive regimen, and individual priorities and goals. In this article, we discuss the top 10 tips in prevention and management of PTLD (Fig. 1).

### Tip 1: Do not use belatacept maintenance immunosuppression in EBV-seronegative recipients

Phase 3 randomized controlled trials (RCTs) (BENEFIT, BENEFIT-EXT) showed a markedly increased and early-onset PTLD risk in EBV-seronegative KTRs treated with belatacept, often involving the central nervous system (CNS) [5–7]. In BENEFIT, three cases of PTLD occurred in belatacept group vs one case in cyclosporine (CsA) group within 2 years [8]. Similarly, in BENEFIT-EXT, five cases occurred in the first 2 years on belatacept (four with CNS involvement) [5]. At 7-year follow-up of BENEFIT-EXT, EBV-seronegative incidence rates (per 100 person-years) were 1.71 in the more intensive (MI) regimen, 5.19 in the less-intensive (LI) regimen, and 0 in the CsA arm. By contrast, incidence rates in EBV-positive patients were low and similar across treatment arms (0.12, 0.25, and 0.14 for MI, LI, and CsA, respectively) [9]. The increased risk of PTLD was also evident in the 10-year follow-up of a Phase 2 randomized trial, where three PTLD cases occurred in the higher-intensity belatacept group versus one in the CsA group [10].

Overall, across trials, EBV-seronegative or EBV-unknown recipients receiving belatacept had a 9-fold higher PTLD rate (8/139) than EBV-seropositive recipients (5/810). These findings led to a black boxed warning and a Risk Evaluation and Mitigation Strategy for belatacept, and Food and Drug Administration (FDA) approval is now limited to EBV-seropositive recipients only.

#### Pitfall

Some PTLD cases observed in the BENEFIT and BENEFIT-EXT trials occurred in the context of higher or more frequent belatacept dosing than the current common practice (5 mg/kg monthly), suggesting that the overall burden of immunosuppression may also contribute to PTLD risk.

### Tip 2: Avoid T-cell depleting induction in EBV-mismatched transplants when immunologic risk allows

Early transplant registry analyses, including both EBV-seropositive and -seronegative KTRs, yielded conflicting results regarding the risk of PTLD with lymphodepleting induction agents [antithymocyte globulin (ATG) or alemtuzumab] versus non-depleting agents (basiliximab) [1, 11–13]. As a result, the routine avoidance of lymphodepleting induction in EBV D+/R− KTRs was not consistently adopted in clinical practice. However, more recent studies focusing specifically on EBV-mismatched patients have demonstrated a significantly increased risk of PTLD with lymphodepleting induction compared to basiliximab. In an analysis of the OPTN database, the adjusted subdistribution hazard ratios (aSHR) for PTLD were 1.98 (95% CI, 1.29–3.04; *P* = .002) for ATG and 1.80 (95% CI, 1.04–3.11; *P* = .04) for alemtuzumab, with no significant difference in risk between the two agents [14]. An even higher risk of PTLD with ATG was observed in a European registry analysis, with a reported hazard ratio of 4.4 (95% CI, 1.8–10.6) [4]. These findings suggest that the risk of PTLD should be carefully considered when selecting induction therapy for EBV-mismatched patients, especially when additional risk factors for post-transplant malignancy are present.

#### Pitfall

Some EBV-mismatched patients may also be highly sensitized or have preformed donor-specific antibodies. In such cases, the choice of induction therapy must balance the risk of PTLD against the increased risk for acute rejection, particularly because treating rejection often requires intensifying immunosuppression (IS), which may further increase the risk of malignancy.

### Tip 3: Consider prospective monitoring of EBV viral load during the first year post-transplant in EBV-seronegative recipients

The AST Infectious Diseases Community of Practice (AST ID COP) recommends EBV viral load (VL) surveillance and preemptive intervention during the first year post-transplant in all EBV-seronegative patients [15]. These recommendations are based on retrospective data and represent weak or low-level evidence.

For EBV D−/R− recipients, monthly VL monitoring is suggested to detect community-acquired infection. By contrast, EBV D+/R− recipients warrant more intensive surveillance, typically every 1–2 weeks. Once EBV viremia is detected, weekly monitoring is recommended during the acute phase, with testing frequency gradually reduced as the VL stabilizes and a ‘set point’ is achieved. Any detectable viremia during primary infection should prompt temporary reduction of immunosuppression (RIS), when feasible, to support EBV-specific adaptive immunity. A trend-based interpretation of EBV-VL, taken in the appropriate clinical context, is more informative that single values given the absence of validated thresholds, inter-laboratory variability, and limited positive predictive value of EBV DNAemia for PTLD.

If VL remains persistently elevated despite RIS, rituximab may be considered as an adjunctive therapy. A prophylactic B-cell depletion with rituximab has been shown in a few retrospective studies to reduce EBV proliferation and prevent PTLD [16–18]. Continued monitoring beyond the first post-transplant year may be appropriate in patients with fluctuating IS, recent rejection episodes, or those who have not yet established a stable VL set point.

#### Pitfall

There are currently no RCTs or prospective data supporting EBV-VL monitoring as a surveillance strategy, nor any comparing preemptive monitoring to placebo. A prospective randomized controlled trial is ongoing to evaluate the efficacy of prophylactic rituximab infusion (375 mg/m^2^, intravenously, in the 7 days prior to transplant for living donor transplant recipients and on day 0 or 1 for deceased donor kidney transplant recipients, KTRs) vs placebo in the prevention of primary EBV infection and PTLD in D+/R− recipients (REPLY trial—ClinicalTrials.gov NCT04989491).

### Tip 4: Do not use antivirals or intravenous immunoglobulins as stand-alone therapy for PTLD prevention or treatment in EBV-mismatched recipients

Meta-analyses and systematic reviews have demonstrated that antiviral prophylaxis using agents such as acyclovir, valacyclovir, ganciclovir, or valganciclovir does not decrease the incidence of PTLD in the high-risk EBV D+/R− patient category [19, 20]. Accordingly, the AST ID COP does not routinely recommend antiviral prophylaxis in this population [15]. Similarly, there is insufficient evidence to support the addition of intravenous immunoglobulins (IVIG) to antivirals for prophylaxis, as RCTs have sown no difference in EBV-VL suppression [15, 21, 22]. Therefore, at present, it is not recommended that antiviral therapy and/or IVIG be used alone for the prevention or treatment of PTLD in the absence of other targeted interventions.

#### Pitfall

In the absence of well-designed prospective RCTs, the potential role of antiviral prophylaxis cannot be entirely excluded. A recent meta-analysis of 22 studies involving 13 498 solid organ transplant recipients (SOTRs) (both EBV-seropositive and seronegative) found that antiviral prophylaxis did significantly reduce the incidence of EBV viremia [risk ratio (RR) 0.69, 95% confidence interval (CI) 0.54–0.88]. It also demonstrated a reduction in PTLD risk among pediatric recipients (RR 0.58, 95% CI 0.43–0.79), KTRs (RR 0.63, 95% CI 0.46–0.87), and recipients of T-cell depleting or steroid-based IS (RR 0.54, 95% CI 0.39–0.74 and RR 0.55, 95% CI 0.41–0.73, respectively) [20]. These findings suggest that antiviral agents may still have a role in select populations; however, the optimal context for their use remains needs further study.

### Tip 5: Complete diagnostic and staging tests in patients with suspected PTLD

When PTLD is suspected, a stepwise diagnostic approach is recommended. In addition to clinical history and physical evaluation, whole blood EBV-VL testing should be performed. The diagnostic gold standard is histopathologic confirmation, by excisional biopsy or core needle biopsy.

Once tissue diagnosis is confirmed, classification should be performed according to the 2022 WHO classification, which divides PTLD into four categories: non‐destructive PTLD (including plasmacytic hyperplasia, infectious mononucleosis, florid follicular hyperplasia), polymorphic PTLD, monomorphic PTLD (B-cell, T-cell, or NK-cell types), and classic Hodgkin lymphoma [23]. Routine studies on biopsy specimens should include hematopoietic lineage determination, EBV genome detection, and immunohistochemical staining for CD20. FDG-PET scan is recommended as standard imaging modality, given its high sensitivity (>85%) and specificity (∼90%) for detecting PTLD [24]. If PET is unavailable, contrast-enhanced CT of the chest, abdomen, and pelvis should be performed [25]. In cases with clinical suspicion of CNS involvement, brain MRI and lumbar puncture are advised.

#### Pitfall

Although whole blood EBV-VL determination is recommended as part of the diagnostic algorithm, it should not be used alone to confirm or exclude a diagnosis of PTLD. For example, in pediatric patients, quantitative VL threshold for the diagnosis of PTLD has 95% negative predictive value but a very poor positive predictive value of 28%, resulting in a significant burden of unnecessary testing [26]. Similarly, in low-risk, EBV-seropositive adult recipients, elevated VL has high specificity for EBV-positive PTLD, but poor sensitivity, failing to detect EBV-negative PTLD.

### Tip 6: Start stepwise therapy for confirmed PTLD

RIS aiming to restore recipient’s immune function is typically the first step in management and can lead on its own to response in 45%–63% of patients, with most achieving a complete response [27, 28]. The best responses are seen in low-risk patients: those with early lesions, early-stage, and non-bulky disease. The optimal approach for RIS is unclear and the risk of acute and chronic rejection must be taken into consideration and communicated to the patient. Typically, in patients with mild or localized disease, antimetabolites are stopped first and calcineurin inhibitors are reduced by ∼30%–50%. Consideration for stopping both calcineurin inhibitors and antimetabolites, while maintaining on prednisone alone, can be made in extensive/bulky disease, in cases of CNS involvement, or in critically ill patients [29, 30]. Limited data from European transplant centers suggest that conversion from CNIs to mTOR inhibitors has been successful in achieving remission [31, 32], however this approach is still not included in the NCCN guidelines [30]. Whether or when to withdraw belatacept for patients with newly diagnosed PTLD and when or how to re-escalate IS after PTLD remission remain critically important, unanswered questions that warrant future research.

Response to RIS is usually seen early (2–4 weeks). Clinical and radiographic evaluation following RIS should determine the indication of targeted therapies [25]. These therapies include initiation of rituximab monotherapy in patients with CD20^+^ B cell PTLD then subsequent CHOP (cyclophosphamide, doxorubicin, vincristine, prednisolone) cytotoxic chemotherapy in relapsed or refractory adult monomorphic CD20^+^ B-cell PTLD [33–37], and R‐CHOP 21, as recommended by the RSST trial [34].

#### Pitfall

Localized disease can sometimes be treated with surgical resection and local radiotherapy along with RIS. Certain special forms of PTLD require additional, disease-specific measures. For example, PTLD with CNS involvement often necessitates the MATRIx regimen (high-dose methotrexate, cytarabine, rituximab, and thiotepa), followed by consolidation with either whole brain radiotherapy or myeloablative chemotherapy with autologous stem cell transplantation. Brentuximab vedotin may be added for CD30^+^ T-cell–type monomorphic PTLD [38]. For Hodgkin lymphoma-type PTLD, immune checkpoint inhibitors may be considered; however, these carry substantial risks, including rejection in 41.2% of cases, immune-related adverse events in 18.5%, and allograft failure in 23.5% [39]. Close monitoring of allograft function is required during RIS including periodic checks of serum creatinine, proteinuria, and donor-derived cell-free DNA.

### Tip 7: Consider enrolling in clinical trials such as EBV-specific cytotoxic T lymphocytes (EBV-CTLs) therapy for relapsed or refractory EBV-positive PTLD

For patients with EBV-positive PTLD who fail to respond or relapse after RIS, rituximab, and standard chemotherapy, EBV-CTLs therapy is evolving as an option to restore EBV-specific cellular immunity. This adoptive cellular therapy selectively targets EBV-infected B cells which drive PTLD. EBV-CTLs can be manufactured from autologous sources (i.e. patients themselves), their original organ donor, or partially HLA-matched third-party donors [40–42]. Phase 2 clinical trials have demonstrated response rates of ∼66% in patients with EBV^+^ refractory PTLD, with most achieving complete or partial remission and minimal associated toxicity [42–45]. More recently, the ALLELE study, the only multi-center Phase 3 trial, reported that 52% (15 of 29) of SOTRs treated with tabelecleucel, an off-the-shelf, allogeneic EBV-specific T-cell immunotherapy, achieved an objective response at a median follow-up of 6 months [41]. No new safety concerns were identified, and the therapy was well tolerated. Tabelecleucel is currently the only allogeneic T-cell therapy approved in the European Union for the treatment of relapsed or refractory EBV + PTLD in adults and children aged 2 years and older. It was also granted marketing authorization by the US FDA in 2022 for use under ‘exceptional circumstances’.

#### Pitfall

Despite its promise, EBV-CTL therapy is limited by technical complexity, high cost, and restricted availability outside of specialized transplant centers. In addition, there is still a lack of randomized controlled studies supporting its widespread use.

### Tip 8: Consider CD19 CAR T-cell therapy in refractory or relapsing EBV-negative PTLD

In KTRs with EBV-negative PTLD that is relapsed or refractory to rituximab and standard chemotherapy, CD19-directed chimeric antigen receptor (CAR) T-cell therapy has emerged as a promising therapeutic option. CAR T cells are genetically engineered to selectively target CD19-expressing lymphoma cells. Clinical experience remains limited, with only 34 cases of PTLD treated with CAR T-cell therapy reported in the literature, of which 24 involving KTRs [46]. Preliminary data suggest that the overall response rate in SOTRs is comparable to that observed in the general population (∼69%), with an acceptable toxicity profile in terms of cytokine release syndrome, immune effector cell-associated neurotoxicity syndrome, and allograft dysfunction (14% risk of allograft loss). Given these findings, CAR T-cell therapy represents a viable therapy in selected patients with refractory PTLD, although close monitoring for treatment-related toxicity and graft function is essential [47–49].

#### Pitfall

Data regarding the efficacy of CAR T-cell therapy in KTRs are still limited to small, highly selected cohorts in expert centers. The main challenge with CAR T-cell therapy in KTRs is the need for extensive monitoring of allograft function and modification of IS during the course of therapy to ensure optimal therapeutic benefit and to mitigate the risk of rejection or allograft loss. IS must be reduced before leukapheresis to facilitate adequate manufacturing of CAR T cells, and again after CAR-T infusion to promote their expansion, persistence, and function. In most cases, this involves withdrawal of both the calcineurin inhibitor and antimetabolite, with maintenance of glucocorticoid. This approach carries a risk of rejection, necessitating vigilant monitoring of allograft function [50].

### Tip 9: Cautiously monitor complications of PTLD treatment

In all major clinical trials evaluating rituximab plus standard CHOP chemotherapy for PTLD, treatment-related toxicity was substantial. The most common severe adverse events were cytopenia and infection. Over one-third of patients experienced grade 3 or 4 infections, most commonly pneumonia, febrile neutropenia, sepsis, and varicella-zoster virus reactivation [34, 51]. Therefore, the AST ID COP has recommended prophylaxis for *Pneumocystis jirovecii* pneumonia during chemo/targeted therapies for PTLD [15]. Another major concern is acute rejection in patient maintained on RIS-containing strategies, with rates as high as 32% in retrospective studies [27].

#### Pitfall

The optimal approach to managing IS during PTLD treatment remains unclear, as does the best method for rejection monitoring. Evidence from KTRs treated with immune checkpoint inhibitors suggests that donor-derived cell-free DNA may have a role in the early detection of allograft rejection [52–54].

### Tip 10: DON’T deny kidney re-transplantation for patients with a history of PTLD

Despite concerns that IS resumption for a kidney re-transplantation may trigger PTLD relapse, several studies have shown that re-transplantation in carefully selected patients with sustained hematological remission is safe [55–58]. In a French cohort of 52 patients who underwent kidney re-transplantation after PTLD, only one patient (1.92%) experienced PTLD recurrence within 24 months of re-transplantation [55]. Similar findings were observed in analyses of the UNOS database. Among 254 patients with a history of PTLD who received a kidney re-transplantation between 2000 and 2019, PTLD recurred in only 2.8% of cases. Outcomes in these patients were comparable to those of second transplant recipients without previous PTLD, with similar 5-year death-censored graft failure (9.5% vs. 12.6% respectively, *P* = .21), all-cause mortality (8.3% vs. 11.8%, *P* = .51), and 1-year acute rejection rates (11.0% vs. 9.3%, *P* = .36) [3]. These findings support the safety and feasibility of kidney re-transplantation in this population.

#### Pitfall

Despite the reassuring safety data, the optimal timing of re-transplantation after PTLD remission remains uncertain. Risk factors for recurrence include early re-transplantation, incomplete remission, high-risk PTLD histology, poor International Prognostic Index score (advanced age, poor performance status, elevated LDH, advanced stage, and multiple extranodal sites), and EBV seronegativity [55, 59]. Therefore, careful patient selection is essential. Most experts recommend delaying re-transplantation for at least 1–2 years after achieving complete remission [59, 60], with thorough confirmation of sustained hematologic remission via physical exam, laboratory tests, and PET or CT imaging of the chest, abdomen, and pelvis. It has also been suggested by some authors to delay re-transplantation until conversion to EBV-seropositive status and confirmation of undetectable EBV-VL [61]. There are currently no established guidelines for monitoring of PTLD recurrence or managing IS after re-transplantation. Induction with IL-2R antagonsits [55], minimization of IS to the lowest effective dose, and close monitoring of EBV DNA levels have been suggested, but individualized risk assessment remains critical.

## CONCLUSION

Our understanding of PTLD has advanced substantially over the past decade, providing multiple strategies for its prevention and management. However, prospective clinical trials are still needed to refine risk-stratified prevention, standardize treatment algorithms, and develop safer approaches to IS. Future research should prioritize validated EBV monitoring strategies, biomarkers predicting treatment response or relapse, optimization of cellular therapies, and evidence-based guidance for IS management and re-transplantation after remission.

## Data Availability

No new data were generated or analyzed in support of this research.

## References

[1] Faull RJ, Hollett P, McDonald SP. Lymphoproliferative disease after renal transplantation in Australia and New Zealand. Transplantation. 2005;80:193–7. 10.1097/01.TP.0000165098.49658.F3 16041263

[2] Caillard S, Lamy FX, Quelen C et al. Epidemiology of posttransplant lymphoproliferative disorders in adult kidney and kidney pancreas recipients: report of the French registry and analysis of subgroups of lymphomas. Am J Transplant. 2012;12:682–93. 10.1111/j.1600-6143.2011.03896.x 22226336

[3] Potluri VS, Zhang S, Schaubel DE et al. The Association of Epstein-Barr virus donor and recipient serostatus with outcomes after kidney transplantation : a retrospective cohort study. Ann Intern Med. 2025;178:157–66. 10.7326/ANNALS-24-00165 39869913 PMC12238997

[4] Ludvigsen LUP, Åsberg A, Spetalen S et al. Risk and prognosis of posttransplant lymphoproliferative disease in Epstein–Barr virus-seronegative kidney transplant recipients—an observational cohort study from Norway and western Denmark. Am J Transplant. 2025;25:1547–60. 10.1016/j.ajt.2025.01.035 39884653

[5] Durrbach A, Pestana JM, Pearson T et al. A phase III study of belatacept versus cyclosporine in kidney transplants from extended criteria donors (BENEFIT-EXT study). Am J Transplant. 2010;10:547–57. 10.1111/j.1600-6143.2010.03016.x 20415898

[6] Vincenti F, Charpentier B, Vanrenterghem Y et al. A phase III study of belatacept-based immunosuppression regimens versus cyclosporine in renal transplant recipients (BENEFIT study). Am J Transplant. 2010;10:535–46. 10.1111/j.1600-6143.2009.03005.x 20415897

[7] Larsen CP, Grinyó J, Medina-Pestana J et al. Belatacept-based regimens versus a cyclosporine A-based regimen in kidney transplant recipients: 2-year results from the BENEFIT and BENEFIT-EXT studies. Transplantation. 2010;90:1528–35. 10.1097/TP.0b013e3181ff87cd 21076381

[8] Vincenti F, Larsen CP, Alberu J et al. Three-year outcomes from BENEFIT, a randomized, active-controlled, parallel-group study in adult kidney transplant recipients. Am J Transplant. 2012;12:210–7. 10.1111/j.1600-6143.2011.03785.x 21992533

[9] Durrbach A, Pestana JM, Florman S et al. Long-term outcomes in belatacept- versus cyclosporine-treated recipients of extended criteria donor kidneys: final results from BENEFIT-EXT, a Phase III randomized study. Am J Transplant. 2016;16:3192–201. 10.1111/ajt.13830 27130868 PMC5516151

[10] Vincenti F, Blancho G, Durrbach A et al. Ten-year outcomes in a randomized phase II study of kidney transplant recipients administered belatacept 4-weekly or 8-weekly. Am J Transplant. 2017;17:3219–27. 10.1111/ajt.14452 28758341 PMC5724691

[11] Kirk AD, Cherikh WS, Ring M et al. Dissociation of depletional induction and posttransplant lymphoproliferative disease in kidney recipients treated with alemtuzumab. Am J Transplant. 2007;7:2619–25. 10.1111/j.1600-6143.2007.01972.x 17868060 PMC2778321

[12] Bustami RT, Ojo AO, Wolfe RA et al. Immunosuppression and the risk of post-transplant malignancy among cadaveric first kidney transplant recipients. Am J Transplant. 2004;4:87–93. 10.1046/j.1600-6135.2003.00274.x 14678038

[13] Dharnidharka VR, Sullivan EK, Stablein DM et al. Risk factors for posttransplant lymphoproliferative disorder (PTLD) in pediatric kidney transplantation: a report of the North American pediatric renal transplant cooperative study (NAPRTCS). Transplantation. 2001;71:1065–8. 10.1097/00007890-200104270-00010 11374404

[14] Attieh RM, Wadei HM, Mao MA et al. The impact of induction therapy on the risk of posttransplant lymphoproliferative disorder in adult kidney transplant recipients with donor-recipient serological Epstein-Barr virus mismatch. Am J Transplant. 2024;24:1486–94. 10.1016/j.ajt.2024.02.028 38447887

[15] Allen UD, Preiksaitis JK, AST Infectious Diseases Community of Practice. Post-transplant lymphoproliferative disorders, Epstein–Barr virus infection, and disease in solid organ transplantation: guidelines from the American society of transplantation infectious diseases community of practice. Clin Transplant. 2019;33:e13652. 10.1111/ctr.13652 31230381

[16] Choquet S, Varnous S, Deback C et al. Adapted treatment of Epstein-Barr virus infection to prevent posttransplant lymphoproliferative disorder after heart transplantation. Am J Transplant. 2014;14:857–66. 10.1111/ajt.12640 24666832

[17] Walti LN, Mugglin C, Sidler D et al. Association of antiviral prophylaxis and rituximab use with posttransplant lymphoproliferative disorders (PTLDs): a nationwide cohort study. Am J Transplant. 2021;21:2532–42. 10.1111/ajt.16423 33289340 PMC8359347

[18] van der Velden WJFM, Mori T, Stevens WBC et al. Reduced PTLD-related mortality in patients experiencing EBV infection following allo-SCT after the introduction of a protocol incorporating pre-emptive rituximab. Bone Marrow Transplant. 2013;48:1465–71. 10.1038/bmt.2013.84 23749107

[19] AlDabbagh MA, Gitman MR, Kumar D et al. The role of antiviral prophylaxis for the prevention of Epstein–Barr virus-associated posttransplant lymphoproliferative disease in solid organ transplant recipients: a systematic review. Am J Transplant. 2017;17:770–81. 10.1111/ajt.14020 27545492

[20] Moghadamnia M, Delroba K, Heidari S et al. Impact of antiviral prophylaxis on EBV viremia and posttransplant lymphoproliferative disorders in solid organ transplant recipients: a systematic review and meta-analysis. Virol J. 2025;22:11. 10.1186/s12985-025-02623-y 39815274 PMC11737057

[21] Humar A, Hébert D, Davies HD et al. A randomized trial of ganciclovir versus ganciclovir plus immune globulin for prophylaxis against Epstein-Barr virus related posttransplant lymphoproliferative disorder. Transplantation. 2006;81:856–61. 10.1097/01.tp.0000202724.07714.a2 16570008

[22] Allen UD, L’Huillier AG, Bollard CM et al. The IPTA Nashville consensus conference on post-transplant lymphoproliferative disorders after solid organ transplantation in children: IV-consensus guidelines for the management of post-transplant lymphoproliferative disorders in children and adolescents. Pediatr Transplant. 2024;28:e14781. 10.1111/petr.14781 38808744

[23] Alaggio R, Amador C, Anagnostopoulos I et al. The 5th edition of the World Health Organization Classification of Haematolymphoid Tumours: lymphoid neoplasms. Leukemia. 2022;36:1720–48. 10.1038/s41375-022-01620-2 35732829 PMC9214472

[24] Montes de Jesus FM, Kwee TC, Kahle XU et al. Diagnostic performance of FDG-PET/CT of post-transplant lymphoproliferative disorder and factors affecting diagnostic yield. Eur J Nucl Med Mol Imaging. 2020;47:529–36. 10.1007/s00259-019-04481-7 31444510 PMC7005092

[25] Shah N, Eyre TA, Tucker D et al. Front-line management of post-transplantation lymphoproliferative disorder in adult solid organ recipient patients—a British Society for Haematology guideline. Br J Haematol. 2021;193:727–40. 10.1111/bjh.17421 33877688

[26] Allen U, Hebert D, Petric M et al. Utility of semiquantitative polymerase chain reaction for Epstein-Barr virus to measure virus load in pediatric organ transplant recipients with and without posttransplant lymphoproliferative disease. Clin Infect Dis. 2001;33:145–50. 10.1086/321806 11418872

[27] Reshef R, Vardhanabhuti S, Luskin MR et al. Reduction of immunosuppression as initial therapy for posttransplantation lymphoproliferative disorder. Am J Transplant. 2011;11:336–47. 10.1111/j.1600-6143.2010.03387.x 21219573 PMC3079420

[28] Tsai DE, Hardy CL, Tomaszewski JE et al. Reduction in immunosuppression as initial therapy for posttransplant lymphoproliferative disorder: analysis of prognostic variables and long-term follow-up of 42 adult patients. Transplantation. 2001;71:1076–88. 10.1097/00007890-200104270-00012 11374406

[29] Transplantation EEGoR . European best practice guidelines for renal transplantation. Section IV: long-term management of the transplant recipient. IV.6.1. Cancer risk after renal transplantation. Post-transplant lymphoproliferative disease (PTLD): prevention and treatment. Nephrol Dial Transplant. 2002;17 Suppl 4:31–3, 35-6. 10.1093/ndt/17.suppl_4.31 12091638

[30] Zelenetz AD, Gordon LI, Abramson JS et al. NCCN guidelines(R) Insights: B-cell lymphomas 3.2025. J Natl Comp Cancer Netw. 2025;23. 10.6004/jnccn.2025.0048. 41067277

[31] Boratyńska M, Wątorek E, Smolska D et al. Anticancer effect of sirolimus in renal allograft recipients with de novo malignancies. Transplant Proc. 2007;39:2736–9. 10.1016/j.transproceed.2007.08.078 18021973

[32] Pascual J. Post-transplant lymphoproliferative disorder—the potential of proliferation signal inhibitors. Nephrol Dial Transplant. 2007;22 Suppl 1:i27–35. 10.1093/ndt/gfm088 17456616

[33] Burns DM, Clesham K, Hodgson YA et al. Real-world outcomes with rituximab-based therapy for posttransplant lymphoproliferative disease arising after solid organ transplant. Transplantation. 2020;104:2582–90. 10.1097/TP.0000000000003183 33104308

[34] Trappe R, Oertel S, Leblond V et al. Sequential treatment with rituximab followed by CHOP chemotherapy in adult B-cell post-transplant lymphoproliferative disorder (PTLD): the prospective international multicentre Phase 2 PTLD-1 trial. Lancet Oncol. 2012;13:196–206. 10.1016/S1470-2045(11)70300-X 22173060

[35] Choquet S, Trappe R, Leblond V et al. CHOP-21 for the treatment of post-transplant lymphoproliferative disorders (PTLD) following solid organ transplantation. Haematologica. 2007;92:273–4. 10.3324/haematol.10595 17296588

[36] Zimmermann H, Reinke P, Neuhaus R et al. Burkitt post-transplantation lymphoma in adult solid organ transplant recipients: sequential immunochemotherapy with rituximab (R) followed by cyclophosphamide, doxorubicin, vincristine, and prednisone (CHOP) or R-CHOP is safe and effective in an analysis of 8 patients. Cancer. 2012;118:4715–24. 22392525 10.1002/cncr.27482

[37] Gonzalez-Barca E, Domingo-Domenech E, Capote FJ et al. Prospective Phase II trial of extended treatment with rituximab in patients with B-cell post-transplant lymphoproliferative disease. Haematologica. 2007;92:1489–94. 10.3324/haematol.11360 18024397

[38] Amengual JE, Pro B. How I treat posttransplant lymphoproliferative disorder. Blood. 2023;142:1426–37. 10.1182/blood.2023020075 37540819 PMC10731918

[39] Portuguese AJ, Tykodi SS, Blosser CD et al. Immune checkpoint inhibitor use in solid organ transplant recipients: a systematic review. J Natl Comp Cancer Netw. 2022;20:406–416.e11. 10.6004/jnccn.2022.7009 35390767

[40] Comoli P, Labirio M, Basso S et al. Infusion of autologous Epstein-Barr virus (EBV)-specific cytotoxic T cells for prevention of EBV-related lymphoproliferative disorder in solid organ transplant recipients with evidence of active virus replication. Blood. 2002;99:2592–8. 10.1182/blood.V99.7.2592 11895798

[41] Mahadeo KM, Baiocchi R, Beitinjaneh A et al. Tabelecleucel for allogeneic haematopoietic stem-cell or solid organ transplant recipients with Epstein–Barr virus-positive post-transplant lymphoproliferative disease after failure of rituximab or rituximab and chemotherapy (ALLELE): a Phase 3, multicentre, open-label trial. Lancet Oncol. 2024;25:376–87. 38309282 10.1016/S1470-2045(23)00649-6

[42] Haque T, Wilkie GM, Jones MM et al. Allogeneic cytotoxic T-cell therapy for EBV-positive posttransplantation lymphoproliferative disease: results of a Phase 2 multicenter clinical trial. Blood. 2007;110:1123–31. 10.1182/blood-2006-12-063008 17468341

[43] Liu J‐Y, Zhang J‐M, Zhan H‐S et al. EBV-specific cytotoxic T lymphocytes for refractory EBV-associated post-transplant lymphoproliferative disorder in solid organ transplant recipients: a systematic review. Transpl Int. 2021;34:2483–93. 10.1111/tri.14107 34510581

[44] Prockop S, Doubrovina E, Suser S et al. Off-the-shelf EBV-specific T cell immunotherapy for rituximab-refractory EBV-associated lymphoma following transplantation. J Clin Invest. 2020;130:733–47. 10.1172/JCI121127 31689242 PMC6994129

[45] Kazi S, Mathur A, Wilkie G et al. Long-term follow up after third-party viral-specific cytotoxic lymphocytes for immunosuppression- and Epstein-Barr virus-associated lymphoproliferative disease. Haematologica. 2019;104:e356–9. 10.3324/haematol.2018.207548 30792197 PMC6669158

[46] Guy P, Marion O, Oberic L et al. CAR T-cell therapy for refractory posttransplantation lymphoproliferative disorder in a kidney transplant patient. Transplant Direct. 2024;10:e1584. 10.1097/TXD.0000000000001584 38414975 PMC10898664

[47] Yamshon S, Gribbin C, Chen Z et al. Efficacy and toxicity of CD19 chimeric antigen receptor T cell therapy for lymphoma in solid organ transplant recipients: a systematic review and meta-analysis. Transplant Cell Thery. 2024;30:73.e1–73.e12. 10.1016/j.jtct.2023.05.018 37279856

[48] McKenna M, Epperla N, Ghobadi A et al. Real-world evidence of the safety and survival with CD19 CAR-T cell therapy for relapsed/refractory solid organ transplant-related PTLD. Br J Haematol. 2023;202:248–55. 10.1111/bjh.18828 37129856

[49] Krishnamoorthy S, Ghobadi A, Santos RD et al. CAR-T therapy in solid organ transplant recipients with treatment refractory posttransplant lymphoproliferative disorder. Am J Transplant. 2021;21:809–14. 10.1111/ajt.16367 33089906

[50] Murakami N, Webber AB, Nair V. Transplant onconephrology in patients with kidney transplants. Adv Chronic Kidney Dis. 2022;29:188–200.e1. 10.1053/j.ackd.2021.09.002 35817526 PMC9326185

[51] Zimmermann H, Koenecke C, Dreyling MH et al. Modified risk-stratified sequential treatment (subcutaneous rituximab with or without chemotherapy) in B-cell post-transplant lymphoproliferative disorder (PTLD) after solid organ transplantation (SOT): the prospective multicentre Phase II PTLD-2 trial. Leukemia. 2022;36:2468–78. 10.1038/s41375-022-01667-1 35974101 PMC9522585

[52] Schenk KM, Deutsch JS, Chandra S et al. Nivolumab + tacrolimus + prednisone ± ipilimumab for kidney transplant recipients with advanced cutaneous cancers. J Clin Oncol. 2024;42:1011–20. 10.1200/JCO.23.01497 38252910 PMC11677297

[53] Hurkmans DP, Verhoeven JGHP, de Leur K et al. Donor-derived cell-free DNA detects kidney transplant rejection during nivolumab treatment. J Immunother Cancer. 2019;7:182. 10.1186/s40425-019-0653-6 31300068 PMC6626432

[54] Lakhani L, Alasfar S, Bhalla A et al. Utility of serial donor-derived cell-free DNA measurements for detecting allograft rejection in a kidney transplant recipient after PD-1 checkpoint inhibitor administration. Transplant Direct. 2021;7:e656. 10.1097/TXD.0000000000001113 33490381 PMC7817285

[55] Caillard S, Cellot E, Dantal J et al. A french cohort study of kidney retransplantation after post-transplant lymphoproliferative disorders. Clin J Am Soc Nephrol. 2017;12:1663–70. 10.2215/CJN.03790417 28818847 PMC5628715

[56] Karras A, Thervet E, Meur YL et al. Successful renal retransplantation after post-transplant lymphoproliferative disease. Am J Transplant. 2004;4:1904–9. 10.1111/j.1600-6143.2004.00562.x 15476493

[57] Leeaphorn N, Thongprayoon C, Chewcharat A et al. Outcomes of kidney retransplantation in recipients with prior posttransplant lymphoproliferative disorders: an analysis of the 2000-2019 UNOS/OPTN database. Am J Transplant. 2021;21:846–53. 10.1111/ajt.16385 33128832

[58] Rouphael B, Lankireddy S, Lazaryan A et al. Outcomes of kidney retransplantation in recipients with prior post-transplant lymphoproliferative disorder. Clin Transplant. 2016;30:60–65. 10.1111/ctr.12659 26497471

[59] Dierickx D, Habermann TM. Post-Transplantation lymphoproliferative disorders in adults. N Engl J Med. 2018;378:549–62. 10.1056/NEJMra1702693 29414277

[60] Johnson SR, Cherikh WS, Kauffman HM et al. Retransplantation after post-transplant lymphoproliferative disorders: an OPTN/UNOS database analysis. Am J Transplant. 2006;6:2743–9. 10.1111/j.1600-6143.2006.01543.x 17049062

[61] Hanto DW. Retransplantation after post-transplant lymphoproliferative diseases (PTLD): when is it safe?. Am J Transplant. 2004;4:1733–4. 10.1111/j.1600-6143.2004.00623.x 15476467

